# Reducing PTSD symptoms through a gender norms and economic empowerment intervention to reduce intimate partner violence: a randomized controlled pilot study in Côte D'Ivoire

**DOI:** 10.1017/gmh.2017.19

**Published:** 2017-11-17

**Authors:** J. Annan, K. Falb, D. Kpebo, M. Hossain, J. Gupta

**Affiliations:** 1 International Rescue Committee, University of Chicago, USA; 2 International Rescue Committee, USA; 3 Medical School of Abidjan, Abidjan, Côte d'Ivoire; 4 London School of Hygiene and Tropical Medicine, London, UK; 5 George Mason University, Fairfax, Virginia, USA

**Keywords:** Armed conflict, gender-based violence (GBV), interventions, intimate partner violence (IPV), mental health, PTSD

## Abstract

**Background.:**

Women living in war-affected contexts face high levels of gender-based violence, including intimate partner violence (Stark & Ager, 2011). Despite well-documented negative consequences, including posttraumatic stress disorder (PTSD) (Garcia-Moreno *et al.* 2006; Steel *et al.* 2009), evidence remains thin regarding intervention effectiveness to mitigate consequences in these settings.

**Methods.:**

This study used a two-armed parallel pilot randomized controlled trial to compare the impact of a group savings only (control) to gender dialogue groups added to group savings (treatment) on women's symptoms of PTSD in northwestern Côte d'Ivoire. Eligible Ivorian women (18+ years, no prior experience with group savings) were invited to participate and 1198 were randomized into treatment groups.

**Results.:**

In the ITT analyses, women in the treatment arm had significantly fewer PTSD symptoms relative to the control arm (*β*: −0.12; 95% CI: −0.20 to −0.03; *p* = 0.005). Partnered women in the treatment arm who had not experienced intimate partner violence (IPV) at baseline had significantly fewer PTSD symptoms than the control arm (*β* = −0.12; 95% CI: −0.21 to −0.03; *p* = 0.008), while those who had experienced IPV did not show significant differences between treatment and control arms (*β* = −0.09; 95% CI: −0.29 to 0.11; *p* = 0.40).

**Conclusions.:**

Adding a couples gender discussion group to a women's savings group significantly reduced women's PTSD symptoms overall. Different patterns emerge for women who experienced IPV at baseline *v.* those who did not. More research is needed on interventions to improve mental health symptoms for women with and without IPV experiences in settings affected by conflict.

## Background

Women living in places affected by war are at high risk of violence both within their communities and their homes. Evidence suggests that gender-based violence (GBV) is more prevalent in fragile and conflict-affected states than in stable settings (Stark & Ager, 2011). While media and policy tend to focus on violence perpetrated by armed actors, women also face high levels of violence perpetrated by husbands and other male partners (i.e. intimate partner violence, hereafter, IPV) in these settings (Annan & Brier, 2010; Stark & Ager, 2011; Hossain *et al.* 2014).

The negative consequences of war-related violence and IPV include social, physical and psychological impacts, such as depression, anxiety and posttraumatic stress disorder (PTSD) (de Jong *et al.* 2003; Garcia-Moreno *et al.* 2006). PTSD among war-affected populations has been well-documented at around 30% (Steel *et al.* 2009). In at least one study, IPV has been shown to be a stronger predictor of probable past-week PTSD than war-related violence (Gupta *et al.* 2014).

Despite the magnitude of the problem, the evidence remains thin regarding intervention effectiveness for treating the mental health consequences of GBV in conflict settings. A 2013 systematic review found only seven evaluations of mental health and psychosocial interventions for survivors of GBV met inclusion criteria. The review found it difficult to draw robust conclusions due to study limitations; none of the studies included random assignment to treatment and only one of the seven studies had a controlled comparison group (Tol *et al.* 2013). More recently, a group randomized controlled trial in the Democratic Republic of Congo (DRC) tested the effectiveness of cognitive processing therapy (CPT) for survivors of sexual violence. Women assigned to CPT showed significant and substantial improvement in symptoms of PTSD, depression, and anxiety, compared with the control group – suggesting that even in settings of ongoing conflict and poverty, recovery from trauma is possible (Bass *et al.* 2013).

In addition to the treatment of symptoms with mental health interventions, a public health approach calls for an emphasis on prevention, a position echoed within the United Nations Sustainable Development Goals and the WHO's Comprehensive Mental Health Action Plan. It is therefore critical to address risk and protective factors associated with mental health in conflict-affected settings. Given the high prevalence in conflict-affected areas, interventions that focus on IPV reduction in these settings may have secondary benefits on mental health symptoms. To date, however, few studies have rigorously tested IPV-focused interventions within conflict-affected settings (Spangaro *et al.* 2013; Ellsberg *et al.* 2015).

Two recent studies have examined the impact of women's economic empowerment on IPV and mental health in conflict-affected settings and yielded mixed findings regarding mental health. A cash grant with business skills training for women in northern Uganda did not reduce IPV or symptoms of depression in the treatment group (Green *et al.* 2016). However, a recently completed trial of a livestock asset transfer intervention in eastern DRC showed significant reductions in symptoms of anxiety and PTSD, although it did not significantly reduce IPV more than the control group (Glass *et al.* 2017). Another study in DRC examined the impact of a savings group for women survivors of sexual violence in DRC on economic well-being and mental health (it did not aim to reduce IPV): it was not effective in reducing symptoms of depression, anxiety or posttraumatic stress (Bass *et al.* 2016). These mixed results suggest that more studies in other contexts are needed.

In this study, we test whether a village savings and loans program plus a gender dialogue group aimed at reducing IPV (Beyene, 2010) was more effective in reducing PTSD symptoms than the economic component alone, among a population of women exposed to conflict and living in poverty in rural Côte d'Ivoire. The addition of a gender dialogue group builds on previous studies that show the combination of economic interventions with those that challenge gender norms as a promising approach to reducing IPV for those who participate (Pronyk *et al.* 2006). The impacts of this combined approach on women's mental health have not previously been tested.

Many women in Côte D'Ivoire have faced the dual burden of both war-related violence and IPV. The first Ivorian Civil War in the West African nation of Côte d'Ivoire targeted civilians with widespread murder, rape, and terrorization in the early 2000s. Armed conflict broke out again in 2010 after the elections, killing thousands and displacing hundreds of thousands. Available estimates of IPV in Côte d'Ivoire are high, with 22.2% of partnered women aged 15–49 reporting past-year physical IPV and 4.6% reporting past-year sexual IPV (Institut National de la Statistique (INS) et ICF International, 2012). In rural Côte d'Ivoire, the contextual conditions of post-conflict recovery, poverty, and gender inequality may make psychological recovery from trauma and the capacity for resilience even more difficult.

The parent study (Gupta *et al.* 2013) found that women in the gender dialogue group reported experiencing less economic abuse and, for those who attended more than 75% of the sessions, physical IPV significantly reduced. In this study, we examine the gender dialogue group's incremental impact on PTSD symptoms on all women who participated in the intervention and then examine impact by baseline IPV status among partnered women.

## Methods

### Study design

This study is a secondary analysis of a two-armed parallel pilot randomized controlled trial implemented by Yale School of Public Health (YSPH) in partnership with Innovations for Poverty Action (IPA) and the International Rescue Committee (IRC). All data were collected between October 2010 and August 2012. A full description and results of original study is published in Gupta *et al.* (2013).

### Participants

The study took place in northwestern rural Côte d'Ivoire. Eligible women were 18 and over and had no prior participation in group savings programs. Both partnered (e.g. married or in a relationship with a male for at least 1 year) and non-partnered (e.g. single, divorced, widowed women not in a relationship with a male for the last year) women were eligible to participate in the IRC program to preserve community social cohesion.

In total 1271 women completed the baseline survey (96% response rate), of which 981 (77.2%) were partnered. After delivery of village savings and loans (VSLA, or group savings) programming, 1198 were randomized into the treatment groups to continue with VSLA only or to also receive a gender dialogue component in addition to VSLA activities. Ten women died during the course of the program. Of the remaining 1188 women, 97.4% (*n* = 1158) had complete baseline mental health data. At endline, 1110 (95.9%) completed full mental health questions and were included in this analysis. Further details and CONSORT diagram are found elsewhere (Gupta *et al.* 2013). Women with no children were more likely to have both missing data and drop out of the intervention; no demographics or baseline IPV or PTSD outcomes were associated with missingness or attrition. The VSLA-only group participants were significantly more likely to drop out of the program.

### Intervention

A complete description of the intervention components is detailed in Gupta *et al.* (2013). The control arm participated in a group savings program (VSLAs).The treatment arm received both VSLA *and* an 8-session gender dialogue group (GDG), which aimed to address household gender inequalities for women and their partners. It was based on the Stages of Change construct of the Transtheoretical Model, (Prochaska & Velicer, 1997) with sessions that focused on the household economy, setting financial goals, budgeting, and dealing with financial stress, while underscoring the importance of non-violence in the home, respect and communication between men and women, and recognition of the important contributions women make to household well-being. The eight GDG sessions were spread out over a 16 week period (i.e. 4 months), where meetings were held once bi-weekly. These GDG sessions met on top of the weekly VSLA sessions. Both arms met once a week for the VSLA only sessions, while the treatment also met bi-weekly for GDG sessions. GDG sessions were designed to last between 1.5 and 2.5 h. Sessions were facilitated by a pair of (one male and one female) IRC field agents per group (one was a GBV field agent while the other was an economic recovery field agent). These IRC field agents were trained on the basics of facilitation, including creating a safe and respectful environment, active listening skills, and effective questioning (Beyene, 2010). Sessions typically began with a recap of the previous session's themes, discussions of the current session's goals, and various activities including skits, group learning exercises, and discussions, as well as assignment of homework.

### Measures

The main outcome measure for this secondary analysis was PTSD symptoms as measured by the Harvard Trauma Questionnaire – PTSD section (Chronbach's alpha = 0.88). The scale asks 16 questions about posttraumatic stress symptoms on a 4-point scale (with 1 denoting ‘Not at all,’ 2 ‘A little,’ 3 ‘Quite a bit,’ and 4 ‘Extremely’). Women reporting an average of 2 points or higher were coded as probable PTSD in accordance with measurement guidelines (Mollica *et al.* 1992). In this paper, PTSD is used interchangeably with probable PTSD based on this measure as we did not have a clinical assessment of PTSD to confirm diagnosis nor did we conduct clinical validation with this population. However, the French version of the scale has previously been found to be reliable and valid among torture survivors in sub-Saharan African countries (de Fouchier *et al.* 2012). The study instrument was adapted from a questionnaire developed by researchers at the London School of Health and Tropical Medicine (Hossain *et al.* 2010). Surveys were translated into Ivorian French and back-translated into English.

### Procedures

Thirty rural villages were selected for inclusion into the trial based on being identified as a priority by the International Rescue Committee (IRC), the implementing agency, and having not previously received economic empowerment programming by the organization. Six villages were excluded due to challenges with mobilizing village leaders and participants, thus yielding a final set of 24 villages. The IRC Côte d'Ivoire field staff met with village leaders and eligible women to introduce the program and study. Women and village leaders were told that all women would receive the economic empowerment program at the same time, while half of the groups would receive an additional discussion group at an earlier time point than others (i.e. a waitlist control). Women were then placed into 47 groups of 15–30 women.

A baseline survey was conducted in October 2010. All groups began VSLA activities in December 2010. However, due to post-election violence that occurred after the baseline survey, randomization to receive the GDG (treatment) in addition to ongoing economic empowerment activities *v.* continuing with economic activities only (comparison) was delayed until September 2011. Based on recommendations by village leaders as a strategy to promote transparency and minimize potential conflicts, random assignment was done via public lottery. IRC staff held a public event in each participating village where each village chief drew the names of groups within each village that would be randomized to receive the treatment. Groups not randomly drawn during the lottery were told that they would receive the discussion group program upon completion of the study. An endline survey was conducted from July to August 2012.

Trained local female research staffs were matched to participants based on language and ethnicity. In private locations, they completed verbal informed consent with participants and verbally administered paper-based surveys and recorded respondents’ responses; survey interviews were conducted in line with WHO ethical and safety guidelines for research on IPV (World Health Organization, 2001). Research staff verbally translated surveys and informed consent into eleven local languages for women as necessary. A list of local medical, legal, and psychosocial support services was given to participants upon survey completion.

Ethical approval was obtained for all study protocols through the Yale University Human Subjects Committee (#1007007040) and Innovations for Poverty Action (506.11 September-003) Human Subjects Committee. Local, Côte d'Ivoire-based approval was obtained by leadership committees from all participating villages.

### Statistical analysis

Descriptive statistics were generated to assess significant differences at baseline between demographics and treatment arm, probable PTSD (cut-off score), and mean number of trauma symptoms. Using a complete case analysis approach, only women who responded to all items of the mental health section were included in the construction of probable PTSD and mean number of trauma symptom scores. Chi-square, *t* tests, or the Kruskal–Wallis tests were used, as appropriate. Statistical significance was set at *p* < 0.05.

Generalized linear mixed models were used to assess the relationships of interest and accounted for individual, group and village clustering. An interaction term between treatment and time was used to test the incremental effectiveness of the intervention. Analysis of intervention effects on mental health outcomes was conducted among all women, and then, as the primary outcome of the parent study was to reduce IPV, analysis was conducted in models stratified by IPV experience at baseline, dividing partnered women into those who had experienced IPV and those who had not.

Intent to treat analysis was undertaken based on treatment assignment. Per protocol analysis for all women was subsequently undertaken and resulted in a three level exposure variable: (1) VSLA + GDG high adherents (women and their male partners or male family member if they were unpartnered participated in at least 75% of the GDG sessions); (2) VSLA + GDG low adherents (women and their male partners/family members who participated in less than 75% of the sessions); and (3) referent group (VSLA only).

## Results

The average age of women participating was 40.5 (s.d.:12.8) years (Table 1). The majority of women were of Yacouba ethnicity (64.1%) and had no formal education (73.0%). Approximately one in five (21.4%) women in the overall sample did not have a partner in the year preceding the baseline assessment.

**Table 1.** Demographics and PTSD symptom status of women, by treatment arm at baseline (*N* = 1188)

Characteristic	Overall	VSLA + GDG	VSLA Only	*p Value*
	% (*n*)/Mean (s.d.)	% (*n*)/Mean (s.d.)	% (*n*)/Mean (s.d.)	
Overall	100.0 (1188)	54.4 (646)	45.6 (542)	
Demographics				
Age of women	40.5 (12.8)	40.5 (12.3)	40.4 (13.3)	0.8
Marital status				
Married	64.6 (767)	65.8 (425)	63.1 (342)	0.7
Living with partner	10.4 (124)	9.8 (63)	11.3 (61)	
Not living with partner	3.6 (43)	3.9 (25)	3.3 (18)	
Unmarried/no partner	21.4 (254)	20.6 (133)	22.3 (121)	
Women's occupation				
Farming only	17.1 (203)	18.1 (117)	15.9 (86)	0.04
Small business only	45.6 (542)	44.1 (285)	47.4 (257)	
Farming and small business	31.1 (370)	33.1 (214)	28.8 (156)	
Other	6.1 (73)	4.6 (30)	7.9 (43)	
Ethnicity				
Yacouba	64.1 (762)	63.0 (407)	65.5 (355)	0.5
Senoufo, Dioula, Guere	6.2 (74)	6.0 (39)	6.5 (35)	
Other	29.6 (352)	31.0 (200)	28.0 (152)	
Religion^a^				
Christian	43.1 (506)	44.0 (281)	41.9 (225)	0.1
Muslim	14.6 (171)	12.5 (80)	17.0 (91)	
Traditional	18.6 (219)	19.9 (127)	17.1 (92)	
Other/none	23.7 (279)	23.5 (150)	24.0 (129)	
Education^b^				
None	73.0 (865)	73.8 (476)	72.0 (389)	0.6
Primary	20.6 (244)	20.5 (132)	20.7 (112)	
Secondary or higher	6.4 (76)	5.7 (37)	7.5 (39)	
PTSD symptoms at baseline				
Probable PTSD cut-off^c^	19.5 (226)	21.0 (133)	17.8 (93)	0.2
Average PTSD score^c^	1.5 (0.5)	1.6 (0.5)	1.5 (0.5)	0.2

a Three women *missing*.

b Two women *missing*.

c 30 women *missing complete data on mental health items*.

Among 1188 women surveyed at baseline and randomized into study arms, 1158 had complete data on mental health items at baseline (97.5%) and 1110 had complete data on mental health items at endline (95.9% of baseline sample). At baseline, women had a mean symptom score of 1.5 (s.d.:0.5) (Table 1) indicating an average response between having symptoms ‘not at all’ and ‘a little bit’. Slightly less than one in five (19.5%) of the women met the cut-off score for probable PTSD. PTSD status at baseline was not significantly different between treatment arm or adherence level at baseline, but did vary by religion, educational status, occupation, and partnership status.

In the ITT analyses, women in the intervention arm were significantly less likely to meet criteria for the probable PTSD cut-off score relative to the comparison arm (OR: 0.61; 95% CI: 0.40–0.93; *p* = 0.02). Statistically significant reductions were also seen in average PTSD symptoms relative to the comparison arm (*β* = −0.12; 95% CI: −0.20 to −0.03; *p* = 0.005). (See Table 2.)

**Table 2.** Intent-to-treat and per protocol associations of incremental effectiveness of GDGs in addition to VSLAs on PTSD and average trauma symptoms among all women

	PTSD cut-off score (⩾2.0 on scale)				Average trauma symptoms^a^			
	Baseline % (*n*)	Endline % (*n*)	Odds ratio (95% CI)	*p*	Baseline mean (s.d.)	Endline mean (s.d.)	*β* (95% CI)	*p*
Overall	19.5 (226/1158)	20.3 (225/1110)	–	–	1.54 (0.51)	1.55 (0.50)	–	–
Intent to treat only								
VSLA only	17.8 (93/524)	22.7 (114/502)	Reference		1.51 (0.48)	1.59 (0.53)	Reference	
VSLA + GDG	21.0 (133/634)	18.3 (111/608)	0.61 (0.40–0.93)	0.02	1.55 (0.53)	1.52 (0.48)	−0.12 (−0.20 to −0.03)	0.005
Per protocol								
VSLA only	17.8 (93/524)	22.7 (114/502)	Reference		1.51 (0.48)	1.59 (0.53)	Reference	
VSLA + GDG low adherence	22.6 (81/358)	23.6 (81/344)	0.78 (0.48–1.23)	0.3	1.58 (0.56)	1.57 (0.52)	−0.09 (−0.18 to 0.007)	0.07
VSLA + GDG high adherence	18.8 (52/276)	11.4 (30/264)	0.40 (0.22–0.71)	0.002	1.52 (0.51)	1.45 (0.42)	−0.15 (−0.25 to −0.05)	0.003

a Number of observations for average trauma symptoms is the same as the PTSD cut-off score observations.

Per protocol analyses revealed that those with high adherence to the program (both the woman and her partner or male family member attended at least 75% of the GDG sessions) showed they were significantly less likely to meet the probable PTSD cut-off score (OR: 0.40; 95% CI: 0.22–0.71; *p* = 0.002) and had a significant reduction on the average symptom scale (*β* = −0.15; 95% CI: −0.25 to −0.05; *p* = 0.003) than the VSLA only group. No significant effects were found for women in the low adherence group.

Among partnered women, for those who experienced IPV at baseline 26.3% met the cut-off for probable PTSD and had an average symptom score of 1.66 (s.d. 0.55). Women who had not experienced IPV at baseline had half the rate of probable PTSD cut-off at 13.1% and had an average symptom score of 1.45 (s.d. 0.45) (Tables 3 and 4).

**Table 3.** Intent-to-treat and per protocol associations of incremental effectiveness of GDGs in addition to VSLAs on PTSD and average trauma symptoms among partnered women who reported any physical and/or sexual IPV at baseline (N = 202)

	PTSD cut-off score (⩾2.0 on scale)				Average trauma symptoms			
	Baseline % (*n*)	Endline % (*n*)	Odds ratio (95% CI)	*p*	Baseline mean (s.d.)	Endline mean (s.d.)	*β* (95% CI)	*p*
Overall	26.3 (53)	23.7 (46)	–	*–*	1.66 (0.55)	1.59 (0.50)	–	*–*
Intent to treat only								
VSLA only	25.0 (22)	25.9 (22)	Reference		1.62 (0.55)	1.60 (0.51)	Reference	
VSLA + GDG	27.2 (31)	22.0 (24)	0.72 (0.29, 1.82)	0.5	1.70 (0.54)	1.59 (0.49)	−0.09 (−0.29, 0.11)	0.4
Per protocol								
VSLA only	25.0 (22)	25.9 (22)	Reference		1.62 (0.55)	1.60 (0.51)	Reference	
VSLA + GDG low adherence	32.3 (20)	24.6 (14)	0.65 (0.22, 1.88)	0.4	1.74 (0.53)	1.63 (0.51)	−0.11 (−0.34, 0.13)	0.4
VSLA + GDG high adherence	21.1 (11)	19.2 (10)	0.85 (0.29, 2.78)	0.8	1.64 (0.55)	1.55 (0.47)	−0.07 (−0.32, 0.17)	0.6

**Table 4.** Intent-to-treat and per protocol associations of incremental effectiveness of GDGs in addition to VSLAs on PTSD and average trauma symptoms among partnered women who did not report any physical and/or sexual IPV at baseline (N = 709)

	PTSD cut-off score (⩾2.0 on scale)				Average trauma symptoms			
	Baseline % (*n*)	Endline % (*n*)	Odds ratio (95% CI)	*p*	Baseline mean (s.d.)	Endline mean (s.d.)	*β* (95% CI)	*p*
Overall	13.1 (93)	17.9 (122)	–	*–*	1.44 (0.44)	1.52 (0.50)	–	*–*
Intent to treat only								
VSLA only	12.6 (40)	20.0 (61)	Reference		1.45 (0.43)	1.57 (0.54)	Reference	
VSLA + GDG	13.6 (53)	16.1 (61)	0.69 (0.38, 1.26)	0.2	1.43 (0.46)	1.49 (0.47)	−0.12 (−0.21 to −0.03)	0.008
Per protocol								
VSLA only	12.6 (40)	20.0 (61)	Reference		1.45 (0.43)	1.57 (0.54)	Reference	
VSLA + GDG low adherence	13.6 (29)	22.5 (47)	1.06 (0.54, 2.10)	0.9	1.44 (0.48)	1.56 (0.52)	−0.01 (−0.12 to 0.10)	0.9
VSLA + GDG high adherence	13.6 (24)	24.7 (14)	0.32 (0.14, 0.73)	0.007	1.42 (0.43)	1.41 (0.40)	−0.14 (−0.26 to −0.02)	0.02

Partnered women who had experienced IPV at baseline and who were in the VSLA + GDG treatment group were less likely to have probable PTSD (OR: 0.72; 95% CI: 0.29–1.82; *p* = 0.5) and had on average lower symptoms (*β* = −0.09; 95% CI: −0.29 to 0.11; *p* = 0.40) than those in the VSLA only (Table 3). Notably, neither association was statistically significant.

For partnered women who had not experienced IPV at baseline, those in the VSLA + GDG treatment group had significantly fewer symptoms on average than those in the VSLA only group (*β* = −0.12; 95% CI: −0.21 to −0.03; *p* = 0.008). They were also less likely to meet the probable PTSD cut-off, however this difference was not significant (OR: 0.69; 95% CI: 0.38–1.26; *p* = 0.2) (Table 3). Per protocol analysis showed that women who had not experienced IPV at baseline who were adherent to the program showed significant effects while no significant effects were found for those in the low adherence group. For women who experienced IPV at baseline, neither low nor high adherents showed significant effects from the VSLA + GDG intervention (Tables 3 and 4).

## Discussion

This study shows that adding a discussion group focused on challenging inequitable gender norms to a savings group overall significantly reduced women's posttraumatic stress symptoms compared with a savings group alone. These findings add to mixed findings from other studies, including a recent economic intervention study in eastern DRC that yielded secondary mental health benefits (Glass *et al.* 2017) as well as studies on two economic interventions in conflict-affected DRC and northern Uganda that did not improve mental health (Bass *et al.* 2016; Green *et al.* 2016).

We find an important difference in how the intervention affected mental health based on whether women experienced or did not experience IPV at baseline. Women who experienced IPV at baseline started with higher PTSD symptoms (and higher percentage above cut-off) at baseline. At the conclusion of the trial, women who reported IPV and who were in the VSLA + GDG group reported decreased PTSD symptoms. For the women who reported IPV at baseline and who were in the VSLA only arm, the levels of PTSD symptoms remained largely unchanged. However, the difference between the two groups was not significant.

A different pattern, however, emerged for women who were not experiencing IPV at baseline. They started with fewer symptoms at baseline, and both treatment and control group reported increased symptoms at endline. However, the increase in PTSD symptoms was significantly smaller for women in the discussion group compared with the savings only group.

The overall increase in symptoms for those who had not experienced IPV at baseline may have been due to post-election violence that occurred during the implementation of the intervention. The conflict in 2010–2011 left approximately 3000 killed, displaced hundreds of thousands throughout the country, and left many in fear of being targeted due to ethnicity or political association. While the savings groups started before the conflict, the discussion groups were delayed until after the conflict and therefore may have supported women in coping with the related exposure to violence and disruption, as demonstrated by the attenuated increase in symptoms. The timing of the discussion groups may have been particularly helpful with their emphasis on optimal utilization of household financial resources following a period marked by heightened economic stress.

Another potential mechanism driving the attenuated increase in symptoms for the non-IPV group is the social support gained through the discussion group, which increased the number of meetings with other women and couples compared with those who only participated in the savings groups. Social support has consistently been found to be a protective factor for PTSD (Ozer *et al.* 2003). While women who only participated in the savings groups discussed increased perceptions of social support in qualitative interviews, the discussion group may have fostered greater social support in the household since it involved couples as opposed to women only. Men's reports from the VSLA + GDG group also indicated increased social support among male participants, which may also contribute to the findings (Falb *et al.* 2014).

We did not see the same overall increase of posttraumatic symptoms in the women with IPV experience, possibly due to their already increased symptom level and their exposure to partner violence. The increased potential exposure to disruption, fear and violence from the conflict did not incrementally increase their symptoms. Our hypothesis was that gender discussion groups with the primary aim of decreasing IPV would also be effective in decreasing PTSD symptoms, due to decreased IPV and improved partner relationships. Despite the lack of statistically significant findings on PTSD symptoms for those with IPV at baseline, the trend points in this direction with those receiving the gender discussion group reporting decreased symptoms. The parent study showed a reduction in economic abuse for those in the discussion treatment group as well as physical violence for those who attended more than ¾ of the sessions (Gupta *et al.* 2013). Qualitative interviews with male partners who participated in the gender dialogue groups also indicate improvements in gender dynamics and lower use of violence in relationships (Falb *et al.* 2014).

While significant effects were observed for women who did not report IPV at baseline, it should be noted that the size of the effect was small (−0.12). This is similar in range to the −0.21 effect size of the economic intervention in DRC (Glass *et al.* 2017), both of which are in the small range for effect sizes, especially in comparison with the CPT intervention conducted in DRC, which had an effect size of 1.4 (Bass *et al.* 2013). However, unlike the CPT intervention study, which targeted women who were above a certain symptom threshhold, the current study did not target a clinical population. More research is needed to examine how economic, gender norms, and mental health interventions improve mental health for women impacted by conflict and how best to target women for interventions in these settings. This study suggests that women experiencing IPV may react differently to violence and disruption related to conflict and consequently may need different or altered interventions; further research is needed to understand this difference.

There are several limitations to the study. The first is that there were limited fidelity measures for the intervention due to conflict that occurred in the midst of the intervention and the challenges of collecting ongoing data. Second, there is a time overlap of the intervention and the recall period of 1-year for the endline in order to make it comparable with the baseline. Third, the study did not have a true control group so we are unable to know the impact of the savings group intervention on its own. Without a pure control group, we are also unable to determine whether the increase in PTSD symptoms was a broader secular trend or whether there was something specific to the savings group that increased symptoms. Fourth, the parent study was not powered to detect changes in PTSD symptoms or subgroup analysis by IPV experience, thus all analyses should be interpreted as secondary in nature. Fifth, there is the potential that participants were resentful of not receiving the discussion groups given the public nature of the randomization process. However, our pilot work and discussions with the field staff underscored that participants were more interested in receiving the savings intervention, which all of the participants received. This, therefore, mitigated the threat of the knowledge of the assignment having a negative effect on the control group. Finally, no clinical cut-off was established and validated with this population.

Despite these limitations, this is one of the first studies to look at the impact of a primary prevention intervention for IPV on PTSD symptoms among women who are affected both by war-violence and IPV and to examine the differential effects for those experiencing IPV. Further research is needed to understand effective interventions for women who face both conflict violence and IPV, to understand the mechanisms through which they work, and the implementation factors that relate to their effectiveness.
